# Improvement of Carbon Tetrachloride-Induced Acute Hepatic Failure by Transplantation of Induced Pluripotent Stem Cells without Reprogramming Factor c-Myc

**DOI:** 10.3390/ijms13033598

**Published:** 2012-03-16

**Authors:** Hua-Ming Chang, Yi-Wen Liao, Chih-Hung Chiang, Yi-Jen Chen, Ying-Hsiu Lai, Yuh-Lih Chang, Hen-Li Chen, Shaw-Yeu Jeng, Jung-Hung Hsieh, Chi-Hsien Peng, Hsin-Yang Li, Yueh Chien, Szu-Yu Chen, Liang-Kung Chen, Teh-Ia Huo

**Affiliations:** 1 Department of Optics and Photonics, National Central University, Chung-Li 32001, Taiwan; E-Mails: huamingchang@gmail.com (H.-M.C.); sychen@dop.ncu.edu.tw (S.-Y.C.); 2 Institute of Oral Biology, School of Dentistry, National Yang-Ming University, Taipei 11217, Taiwan; E-Mails: rabbity18@yahoo.com.tw (Y.-W.L.); henlichen@nycu.edu.tw (H.-L.C.); 3 Institute of Pharmacology, National Yang-Ming University, Taipei 11217, Taiwan; E-Mails: guchiang@gmail.com (C.-H.C.); ylchang@vghtpe.gov.tw (Y.-L.C.); 4 Division of Urology, Department of Surgery, Taipei Veterans General Hospital, Su-Ao/Yuan-Shan Branch, Yilan County 26444, Taiwan; E-Mails: jsy202@mail.ysvh.gov.tw (S.-Y.J.); alainjhh@yahoo.com.tw (J.-H.H.); 5 School of Medicine, National Yang-Ming University, Taipei 11217, Taiwan; E-Mails: chenyj@vghtpe.gov.tw (Y.-J.C.); d49405004@gmail.com (Y.-H.L.); chpeng1008@gmail.com (C.-H.P.); lihy@vghtpe.gov.tw (H.-Y.L.); lkchen2@vghtpe.gov.tw (L.-K.C.); 6 Department of Obstetrics and Gynecology, Taipei Veterans General Hospital, Taipei 11217, Taiwan; 7 Department of Medical Research and Education, Taipei Veterans General Hospital, Taipei 11217, Taiwan; 8 Department of Ophthalmology, Shin Kong Wu Ho-Su Memorial Hospital, Taipei 11101, Taiwan; 9 School of Medicine, Fu-Jen Catholic University, Taipei 24352, Taiwan; 10 Center for Geriatrics and Gerontology, Taipei Veterans General Hospital, Taipei 11217, Taiwan; 11 Division of Gastroenterology, Department of Internal Medicine, Taipei Veterans General Hospital, Taipei 11217, Taiwan

**Keywords:** induced pluripotent stem cell, c-Myc, carbon tetrachloride, hepatic failure, hepatic encephalopathy

## Abstract

The only curative treatment for hepatic failure is liver transplantation. Unfortunately, this treatment has several major limitations, as for example donor organ shortage. A previous report demonstrated that transplantation of induced pluripotent stem cells without reprogramming factor c-Myc (3-genes iPSCs) attenuates thioacetamide-induced hepatic failure with minimal incidence of tumorigenicity. In this study, we investigated whether 3-genes iPSC transplantation is capable of rescuing carbon tetrachloride (CCl_4_)-induced fulminant hepatic failure and hepatic encephalopathy in mice. Firstly, we demonstrated that 3-genes iPSCs possess the capacity to differentiate into hepatocyte-like cells (iPSC-Heps) that exhibit biological functions and express various hepatic specific markers. 3-genes iPSCs also exhibited several antioxidant enzymes that prevented CCl_4_-induced reactive oxygen species production and cell death. Intraperitoneal transplantation of either 3-genes iPSCs or 3-genes iPSC-Heps significantly reduced hepatic necrotic areas, improved hepatic functions, and survival rate in CCl_4_-treated mice. CCl_4_-induced hepatic encephalopathy was also improved by 3-genes iPSC transplantation. Hoechst staining confirmed the successful engraftment of both 3-genes iPSCs and 3-genes iPSC-Heps, indicating the homing properties of these cells. The most pronounced hepatoprotective effect of iPSCs appeared to originate from the highest antioxidant activity of 3-gene iPSCs among all transplanted cells. In summary, our findings demonstrated that 3-genes iPSCs serve as an available cell source for the treatment of an experimental model of acute liver diseases.

## 1. Introduction

Acute hepatic failure (AHF) is a severe liver injury accompanied by sustained liver damage and complications including increased blood levels of hepatic enzymes and hepatic encephalopathy. Management of severe AHF is one of the most challenging problems in clinical medicine [1]. Liver transplantation has been shown to be the only curative treatment for hepatic failure. Unfortunately, several major limitations of this treatment exist, such as donor organ shortage and high cost [2]. Cell-based therapy has been implicated in the treatment of liver diseases. For example, embryonic stem cells (ESCs) can differentiate into functional hepatocytes that effectively replace injured parenchyma [3].

Without the ESC-related disadvantages, which include ethical issues and immunorejection, induced pluripotent stem cell (iPSC) is an alternative potential cell source for cell-based therapy against liver diseases. iPSCs can be generated from mouse or human fibroblasts using retroviral transfection of four transcription factors (Oct4/Sox2/Klf4/c-Myc) [4–6], and similarly, these 4-genes iPSCs have been shown to give rise to functional hepatocyte-like cells (iPSC-Heps) [7,8]. Moreover, hepatocytes generated from patient-specific iPSCs could be used for the modeling of inherited metabolic disorders [9]. Since teratoma formation from pluripotent stem cells is considered an unacceptable obstacle for the application of stem cell therapy [10,11], our recent study demonstrated that iPSCs without reprogramming factors c-Myc (3-factors iPSCs; introduced with only three factors (Oct4/Sox2/Klf4)) can differentiate into hepatocyte-like cells (3-genes iPSC-Heps) and significantly improve thioacetamide (TAA)-induced acute liver injury with low incidence of tumorigenicity [12]. Although we provided evidence that 3-genes iPSCs may serve as a useful cell source for cell therapy against TAA-induced acute liver disease, most of this data was still premature and whether 3-genes iPSCs rescued an experimental model of AHF, induced by other chemicals, and improved hepatic encephalopathy remained uncertain.

In the present study, we evaluated the hepatoprotective property of 3-genes iPSC transplantation in a carbon tetrachloride (CCl_4_)-induced AHF model in mice. Firstly, we demonstrated that 3-genes iPSCs possess potential to differentiate into functional iPSC-Heps using a stepwise protocol as described previously [12]. 3-genes iPSCs exhibited a remarkable antioxidant activity that prevented CCl_4_-induced cell death *in vitro*. Next, we transplanted 3-genes iPSCs or 3-genes iPSC-Heps into CCl_4_-treated mice via an intraperitoneal route, and then investigated the hepatoprotective effect of 3-genes iPSC transplantation in such a model. Our findings revealed that both 3-genes iPSCs and 3-genes iPSC-Heps were mobilized to the injured livers and effectively reduced hepatic necrotic area and oxidative stress, improved hepatic functions, the survival rate, as well as impaired motor functions in mice pre-treated with CCl_4_.

## 2. Results and Discussion

### 2.1. *In Vitro* Differentiation of iPSCs into iPSC-Heps

**Figure 1:**
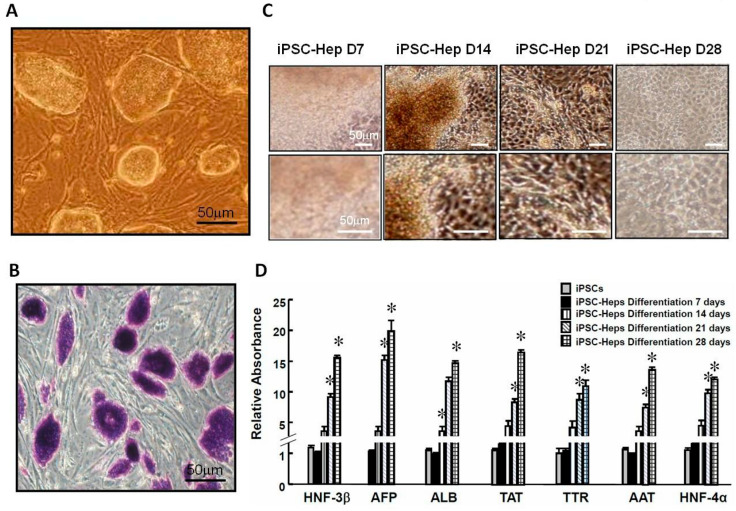
Differentiation of 3-genes iPSCs (induced pluripotent stem cells) into 3-genes iPSC-Heps. (**A**) 3-genes iPSCs are capable of forming colonies similar in appearance to mouse embryonic stem cells (ESCs) and 4-genes iPSCs; (**B**) Colonies of 3-genes iPSCs stained positive for alkaline phosphate; (**C**) Morphological changes of 3-genes iPSC-Heps during hepatocyte differentiation; (**D**) Recruitment of hepatocyte-specific genes, including HNF-3β, AFP, ALB, TTR, AAT, TAT, and HNF-4α during the course of hepatocyte differentiation. In panels A, B and D, scale bar = 50 μm. In panel D, data shown here are the mean ± SD of three independent experiments. * *P* < 0.05 *vs*. undifferentiated iPSCs at Day 0.

**Figure 2:**
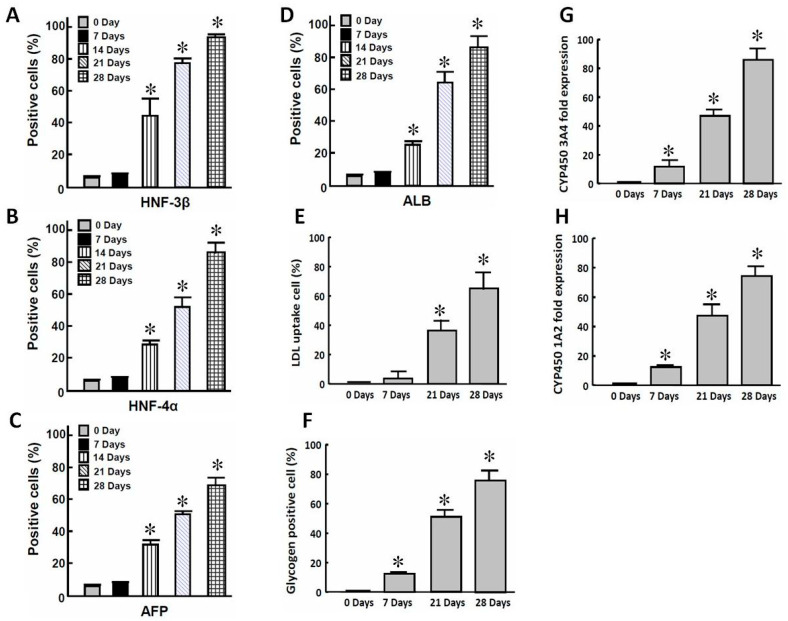
Recruitment of hepatocyte-specific markers, biological functions and enzyme activities. (**A**–**D**) Detection of hepatocyte-specific proteins using immunofluorescence. Immunostaining imaging (800×) results showed several hepatocyte-specific markers, including HNF-3β, AFP, ALB, and HNF-4α; (**E**) The uptake of Low-Density Lipoprotein (LDL) was measured by the incorporation rate of DiI-Ac-LDL and (**F**) the presence of stored glycogen was determined by PAS staining. The relative activities of (**G**) cytochrome P_450_ enzyme CYP3A4 and (**H**) CYP1A2 were analyzed by a commercial ELISA kit. Data shown here are the mean ± SD of three independent experiments. In panels A–H; * *P* < 0.05 *vs*. undifferentiated iPSCs at Day 0.

Recently, we demonstrated that 3-genes iPSCs are similar to both 4-genes iPSCs (iPSCs generated using four conventional reprogramming factors Oct4/Sox2/Klf4/c-Myc) and ESCs in morphology, stem cell markers, and genomic traits [12]. In this study, 3-genes iPSCs were routinely cultured on inactivated MEF monolayers. Consistent with a previous report [12], 3-genes iPSCs are capable of forming colonies similar in appearance to mouse ESCs and 4-genes iPSCs (Figure 1A), and were stained positive for alkaline phosphate (Figure 1B). To further investigate the pluripotent property of 3-genes iPSCs, we investigated the ability of 3-genes iPSCs for embryoid body (EB) formation and for tri-dermal differentiation. Using a different differentiation protocol, 3-genes iPSC-derived EBs could be differentiated into neuron-like cells, osteocyte-like cells, adipocyte-like cells, and regular-beating cardiomyocyte-like cells (ectoderm and mesoderm; data not shown). To evaluate the potential of hepatic-specific differentiation (endoderm), 3-genes iPSC-derived EBs (EB; Figure 1C, upper left) were shifted to hepatic differentiation media, and these cells gradually exhibited more spread and cuboidal morphology over time and eventually differentiated into iPSC-derived hepatocyte-like cells (3-genes iPSC-Heps; Figure 1C; 28 days), as previous reported [12]. Moreover, the gene expressions of several hepatic-specific markers, including HNF-3β, AFP, ALB, TTR, AAT, TAT and HNF-4α were largely increased over time and reached maximal expression at post-differentiation Day 28, indicating the maturation of these hepatocyte-like cells (Figure 1D; * *P* < 0.05 *vs*. undifferentiated iPSCs at Day 0). Along such a differentiation course, immunofluorescence analysis also confirmed the elevation of the protein content of hepatic-specific markers including HNF-3β, HNF-4α, AFP and ALB (Figure 2A–D; *P* < 0.05 *vs*. undifferentiated iPSCs at Day 0).

To further examine whether these 3-genes iPSC-Heps also exhibited hepatic biological functions, we evaluated the hepatic biological functions in these cells, including LDL uptake, glycogen synthesis, and the enzyme activities of the liver enzyme cytochrome P_450_ 3A4 and 1A2 (abbreviated as CYP450 3A4 and CYP450 1A2, respectively; * *P* < 0.05 *vs*. undifferentiated iPSCs at Day 0). As shown in Figure 2, these 3-genes iPSC-Heps acquired ability for LDL uptake and glycogen synthesis (Figure 2E,F, respectively; * *P* < 0.05 *vs*. undifferentiated iPSCs at Day 0), and also exhibited high activity of liver enzyme CYP450 3A4 and CYP450 1A2 (Figure 2G,H, respectively; * *P* < 0.05 *vs*. undifferentiated iPSCs at Day 0), along the course of hepatocyte-specific differentiation.

### 2.2. 3-Genes iPSCs Possess an Antioxidant System Against CCl4-Induced Cell Death *in Vitro*

**Figure 3:**
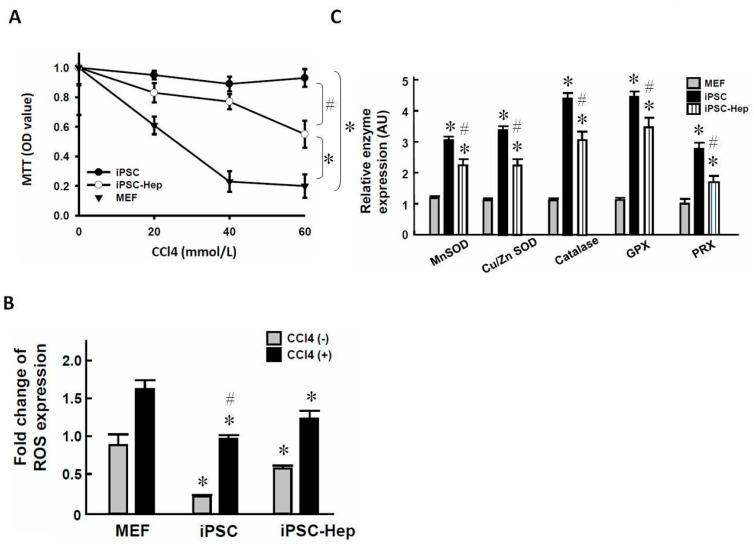
Effect of CCl_4_ administration on reactive oxygen species (ROS) production and cell survival *in vitro*. (**A**) Evaluation of CCl_4_-induced cytotoxicity and (**B**) ROS elevation in MEFs, 3-genes iPSCs, and 3-genes iPSC-Heps; (**C**) Comparison of the gene expression of the antioxidant enzymes in MEFs, 3-genes iPSCs, and 3-genes iPSC-Heps using quantitative RT-PCR. Data shown here are the mean ± SD of three independent experiments. In panels A and C, * *P* < 0.05 *vs*. MEFs. # *P* < 0.05 *vs*. iPSC-Heps. In panel B, * *P* < 0.05 *vs*. MEFs with corresponding treatment. # *P* < 0.05 *vs*. iPSC-Heps with corresponding treatment.

A previous report demonstrated that the mechanism of CCl_4_-mediated hepatotoxicity involves reductive dechlorination of CCl_4_ to a trichloromethyl radical (^•^CCl_3_) which subsequently precipitates membrane lipid peroxidation and hence liver damage [13]. Notably, iPSCs have been shown to possess anti-oxidant ability to suppress oxidative insult [14]. Prior to examining the hepatoprotective activity of 3-genes iPSC and/or 3-genes iPSC-Heps in CCl_4_-treated mice *in vivo*, we evaluated whether 3-genes iPSC and/or 3-genes iPSC-Heps were resistant to CCl_4_-induced cell death. CCl_4_ led to the death of MEFs in a dose-related manner, and the maximal cytotoxic effect was observed at 40 mmol/L CCl_4_ (Figure 3A). Notably, the cytotoxic effect of CCl_4_ was partially blunted by 3-genes iPSC-Heps, and was fully prevented by 3-genes iPSCs (Figure 3A; * *P* < 0.05 *vs*. MEFs; # *P* < 0.05 *vs*. iPSC-Heps). We then compared the ability of reactive oxygen species (ROS) production among MEFs, 3-genes iPSC-Heps, and 3-genes iPSCs, in the presence or absence of CCl_4_. The intracellular ROS levels were the highest in MEFs, slightly lower in 3-genes iPSC-Heps and the lowest in 3-genes iPSCs (Figure 3B, *P* < 0.05 *vs*. MEFs without CCl_4_), indicating the prominent ROS scavenging activity in such cells. As expected, addition of CCl_4_ elicited a rapid increase in ROS production among MEFs, 3-genes iPSCs, and 3-genes iPSC-Heps (Figure 3B, *P* < 0.05 *vs*. MEFs with CCl_4_). Quantitative RT-PCR revealed that the highest expression of several antioxidant enzymes including Mn SOD, Cu/Zn SOD, catalase, GPX, and PRX in undifferentiated 3-genes iPSCs, as compared with 3-genes iPSC-Heps and MEFs (Figure 3C, * *P* < 0.05 *vs*. MEFs; # *P* < 0.05 *vs*. iPSC-Heps). In contrast, the expression of these enzymes was the lowest in MEFs (Figure 3C, * *P* < 0.05 *vs*. iPSCs and iPSC-Heps). These findings showed that the remarkable antioxidant activity of 3-genes iPSCs was associated with the high expression of several antioxidant enzymes.

### 2.3. Intraperitoneal Transplantation of 3-Genes iPSCs Reduced Hepatic Necrotic Area and Oxidative Stress

**Figure 4:**
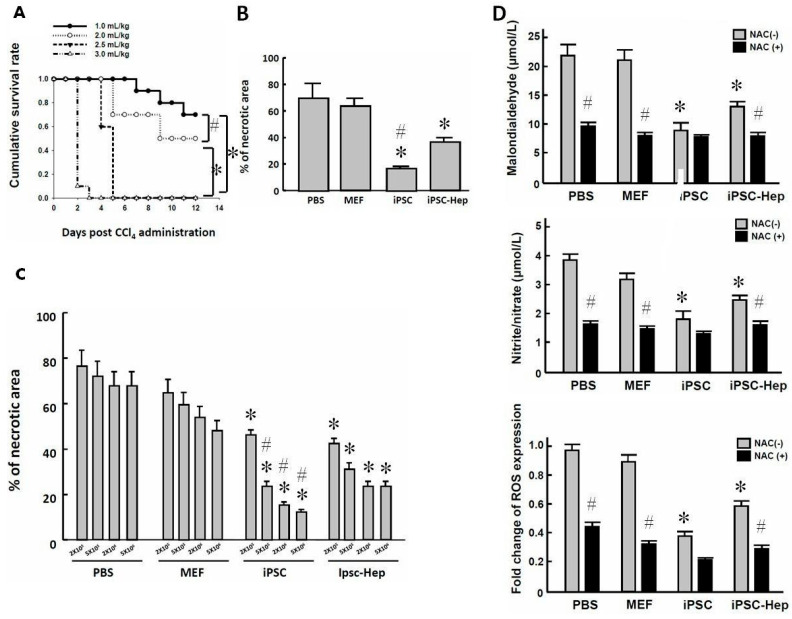
Effect of intraperitoneal cell transplantation (PBS, mouse embryonic fibroblasts (MEFs), iPSCs, or iPSC-Heps) on the hepatic pathology in CCl_4_-treated mice. (**A**) Determination of the optimal dose for the induction of acute hepatic failure (AHF) by intraperitoneal administration of CCl_4_ into BALB/c nude mice. 2.5 mL/kg body CCl_4_ was used as the standard treatment; (**B**) Quantification of H&E-stain of CCl_4_-treated liver tissue from recipients; (**C**) Quantification of H&E-stained liver sections from CCl_4_-treated mice that received PBS injection, transplantation of MEFs, 3-genes iPSCs, or 3-genes iPSC-Heps at 2 × 10^5^, 5 × 10^5^, 2 × 10^6^, and 5 × 10^6^ cells /kg body wt; (**D**) Changes in ROS levels (left), MDA content (middle), and nitrite/nitrate content (right) in the CCl_4_-injured livers of recipients in all groups with and without *N*-acetyl-cysteine (NAC) pretreatment. Data shown here are the mean ± SD of six independent experiments. In panel A, * *P* < 0.05 *vs*. 2.5 mL/kg body wt, # *P* < 0.05 *vs*. 2.0 mL/kg body wt. In panel B, * *P* < 0.05 *vs*. PBS or MEFs. # *P* < 0.05 *vs*. iPSC-Heps. In panel C, * *P* < 0.05 *vs*. PBS or MEFs at the same cell doses. # *P* < 0.05 *vs*. iPSC-Heps at the same cell doses. In panel D, * *P* < 0.05 *vs*. PBS or MEFs, # *P* < 0.05 *vs*. corresponding to recipient with NAC treatment.

**Figure 5:**
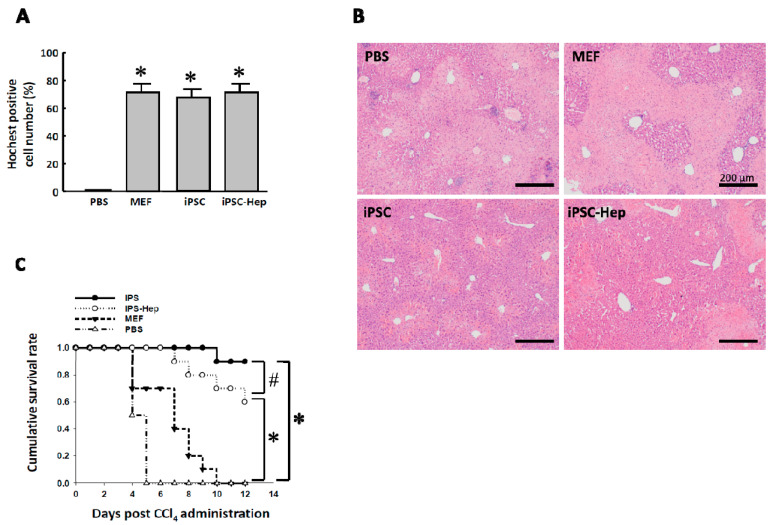
Effect of intraperitoneal cell transplantation (PBS, MEFs, 3-genes iPSCs, or 3-genes iPSC-Heps) on hepatic pathology, survival rate and biochemical parameters in CCl_4_-treated recipients. (**A**) Hoechst-stained cell engraftment in the CCl_4_-injured liver from recipients of PBS, MEFs, iPSCs, or iPSC-Heps; (**B**) Representative H & E stain of CCl_4_-treated liver tissue 24 h after receiving iPSCs, iPSC-Heps, MEFs or PBS treatment; (**C**) Intraperitoneal transplantation of 3-genes iPSCs or 3-genes iPSC-Heps rescued mice from lethal AHF. Data shown here are the mean ± SD of six independent experiments. In panel, * *p* < 0.05 vs. PBS. In panel C, * *p* < 0.05 vs. PBS or MEFs. # *p* < 0.05 vs. iPSC-Heps.

**Figure 6:**
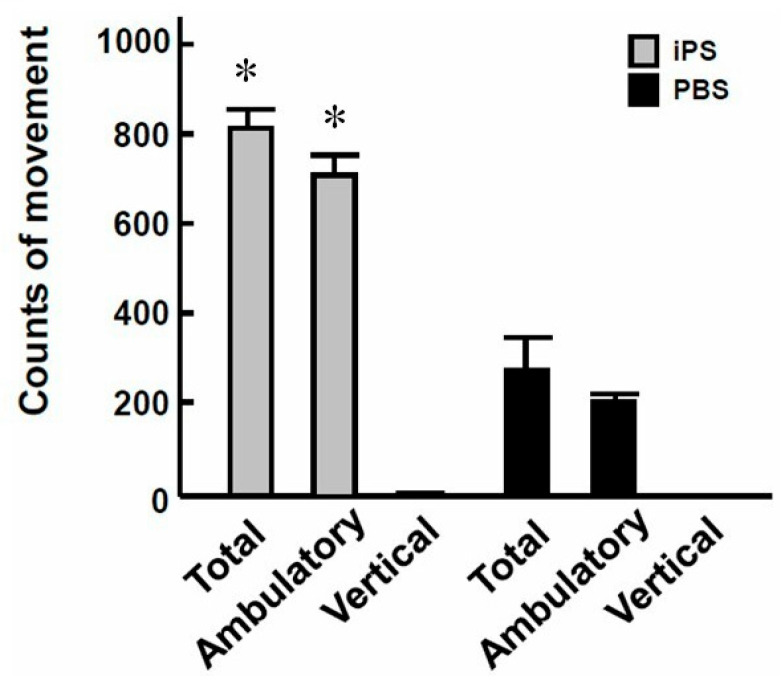
Effect of intraperitoneal cell transplantation on motor activity in CCl_4_-treated mice and tumorigenesis. CCl_4_-treated mice were injected with PBS or transplanted with 3-genes iPSCs (2 × 10^6^ cells) via an intraperitoneal route to determine motor movements. Motor activities in an open field were determined by using the Opto-Varimex animal activity meter. Data are the mean ± SD of six independent experiments. * *P* < 0.05 *vs*. PBS.

AHF is a severe liver disease accompanied by high mortality and hepatic encephalopathy that causes multiorgan failure. Given that 3-genes iPSCs possess prominent antioxidant properties, we subsequently transplanted 3-genes iPSCs, 3-genes iPSC-Heps, MEFs, or injected PBS via an intraperitoneal route, and then assessed the therapeutic potential of these cells in CCl_4_-treated recipients. Firstly, we investigated the doses of CCl_4_ required for induction of *in vivo* lethality in BALB/c nude mice (Figure 4A). We found that CCl_4_ doses greater than 2.5 mL/kg body wt by intraperitoneal injection elicited hyperacute injuries, leading to rapid death (10/10) within five days (Figure 4A). In the remaining experiments, we then used the dose 2.5 mL/kg body wt as the standard treatment (Figures 4–6). Twenty-four hours after cell transplantation at cell dose 2 × 10^6^ cells /kg body wt, histological examination revealed that 3-genes iPSCs exhibited the most pronounced rescuing effect on hepatic necrotic areas rather than other cells and PBS (Figure 4B, * *P* < 0.05 *vs*. PBS or MEFs; # *P* < 0.05 *vs*. iPSC-Heps). The effect of MEF transplantation was not obvious at cell dose 2 × 10^5^, 5 × 10^5^, 2 × 10^6^ and 5 × 10^6^ cells/kg body wt, whereas transplantation of either 3-genes iPSCs or 3-genes iPSC-Heps led to a remarkable reduction in necrotic area in CCl_4_-induced AHF at a cell dose greater than 5 × 10^5^ cells (Figure 4C, * *P* < 0.05 *vs*. MEFs at corresponding cell dose). Notably, the hepatoprotective effect of undifferentiated 3-genes iPSCs was dose-dependent and was higher than that of 3-genes iPSC-Heps (Figure 4C, # *P* < 0.05 *vs*. iPSC-Heps at corresponding cell dose). Along with the antioxidant properties of 3-genes iPSCs and 3-genes iPSC-Heps *in vitro* (Figure 3), we subsequently examined whether transplantation of 3-genes iPSCs or 3-gene iPSC-Heps reduced the production of oxidative substances *in vivo*. Both MDA and nitrate/nitrite are indicative of oxidative damage. CCl_4_ treatment induced the production of MDA, nitrate/nitrite and ROS in livers from all recipients (Figure 4D). The CCl_4_-induced production of MDA, nitrate/nitrite and ROS were not affected by MEF transplantation. Notably, this CCl_4_-induced production of MDA, nitrate/nitrite and ROS was significantly lower in livers from recipients of 3-genes iPSCs and 3-genes iPSC-Heps, compared with PBS or MEFs (* *P* < 0.05 *vs*. PBS or MEFs). *N*-acetyl-cysteine (NAC) is known as an antioxidant that minimizes oxidative stress. Addition of NAC suppressed the CCl_4_-induced elevation of MDA, nitrate/nitrite and ROS in recipients of PBS or MEFs the most and moderately in 3-genes iPSC-Heps (Figure 4D, # *P* < 0.05 *vs*. corresponding recipients with NAC treatment). NAC addition did not lead to further suppression of these low levels of MDA, nitrate/nitrite and ROS in CCl_4_-injured livers from 3-genes iPSC recipients. Remarkably, the elevation of these oxidative substances was consistently maintained by 3-genes iPSCs at a low degree similar to those of MEF or 3-genes iPSC-Hep recipients treated with NAC, indicating the prominent antioxidant activity by 3-genes iPSCs. Compared with that of 3-genes iPSCs, the *in vivo* antioxidant activity of 3-genes iPSC-Heps was relatively lower. These findings demonstrated that 3-genes iPSCs and iPSC-Heps potentially suppressed ROS production and activated antioxidant enzymes in CCl_4_-injured livers.

### 2.4. 3-Genes iPSCs and 3-Genes iPSC-Heps Rescued the Survival of CCl4-Induced AHF

Our previous report revealed that intrasplenic transplantation led to event extent of engraftment among recipients of 3-genes iPSCs, 3-genes iPSC-Heps and MEFs [12]. In this study, we performed a tract-tracing study using immunofluorescence Hochest staining to trace the distribution of the cells, and we observed that MEF, 3-genes iPSCs, and 3-genes iPSC-Heps, with cell dose of 2 × 10^6^ cells by intraperitoneal transplantation, could also mobilize to the CCl_4_-damaged liver area to a similar extent (Figure 5A). Under this condition, 3-genes iPSCs/3-genes iPSC-Heps consistently reduced the hepatic necrotic area, and 3-genes iPSCs gave better efficacy than 3-genes iPSC-Heps at fixed dose of 2 × 10^6^ cells (Figure 5B, a representative H & E stain of CCl4-treated liver tissue 24 h after receiving iPSCs, iPSC-Heps, MEFs or PBS treatment). CCl_4_ injection resulted in lethality in all of the PBS-treated recipients within 5 days, and MEF-treated recipients within 9 days (Figure 5C). However, sixty percent of mice were rescued at Day 12 by transplantation of 3-genes iPSC-Heps (6/10 mice, Figure 5C). Strikingly, transplantation of 3-genes iPSCs rescued ninety percent of mice (9/10 mice, Figure 5C) within the same experimental period.

In PBS-treated mice, hepatic biochemical parameters including alanine aminotransferase (ALT), aspartate aminotransferase (AST) and total bilirubin (TBIL) gradually declined within 72 h, indicating tissue repair in the CCl_4_-injured liver (Table 1). 3-genes iPSC-Heps transplantation led to significant reduction in these biochemical parameters, as compared with PBS-treated or MEF recipients (Table 1; * *P* < 0.05 *vs*. MEFs). Identical to the protective effect on the hepatic necrotic area, the transplantation of 3-genes iPSCs led to an even more pronounced reduction in biochemical parameters than for 3-genes iPSC-Heps or MEFs (Table 1; # *P* < 0.05 *vs*. iPSC-Heps).

### 2.5. 3-Genes iPSCs Improved CCl4-Induced Hepatic Encephalopathy and Reduced Tumorigenesis

Furthermore, we examined whether 3-genes iPSC transplantation could improve hepatic encephalopathy by assessing the motor activity of CCl_4_-treated mice after cell transplantation. Motor activity assay indicated that, 72 h after CCl_4_ administration, the total movements and ambulatory movements were significantly increased in 3-genes iPSC recipients compared to mice which had received PBS injection only (Figure 6A, * *P* < 0.05 *vs*. PBS). These results demonstrated 3-genes iPSC treatment not only improved hepatic functions and animal survival, but also improved CCl_4_-induced hepatic encephalopathy. Furthermore, we performed an extended follow-up study for 4 months and found that recipients of 3-genes iPSCs and 3-genes iPSC-Heps survived, and no tumor formation was observed in either recipients. Severe tumor-like formation was found only in CCl_4_-treated recipients of 4-genes iPSCs (with the oncogene c-Myc), which were used as the positive control of tumorigenicity in this extended study (Table 2). This raised the possibility that obviation of c-Myc in iPSCs and iPSC-Hep may substantially reduce the incidence of tumorigenesis after transplantation.

### 2.6. Discussion

The only curative treatment for hepatic failure is liver transplantation [1]. Unfortunately, this treatment has several major limitations, thus it is urgent to develop an efficient alternative strategy for the treatment of hepatic failure or other acute hepatic diseases. Cell therapy which has minimal invasive procedures and fewer surgical complications has been considered as a potential therapeutic alternative for treatment of liver diseases [15–17]. Although ESCs and iPSCs have been implicated in cell therapy against liver diseases [3,7–9], teratoma formation from pluripotent stem cells is considered as an unacceptable obstacle for stem cell therapy [10,11]. Interestingly, the absence of the reprogramming factor c-Myc was reported to be associated with low incidence of tumorigenicity in our recent study [12] and the study reported elsewhere [18]. In the present study, our data also revealed the successful reprogramming of iPSCs from MEFs without introducing the factor c-Myc (Figure 1), the efficiency of hepatic specific differentiation into 3-genes iPSC-Heps with hepatic-specific genes and proteins (Figure 2), as well as mature biological functions such as LDL uptake and cytochrome p450 activity (Figure 2). In addition, 3-genes iPSCs exhibited a prominent antioxidant system and were resistant to CCl_4_-induced cell death *in vitro* (Figure 3). To ensure the hepatoprotective property of 3-genes iPSCs and 3-genes iPSC-Heps *in vivo*, we tested the hepatoprotective effect of 3-genes iPSCs/3-genes iPSC-Heps after cell transplantation into the mouse model of AHF, induced by the hepatotoxin CCl_4_. Transplantation of 3-genes iPSCs or 3-genes iPSC-Heps effectively rescued these CCl_4_-treated mice from lethal AHF, potentially reduced hepatic necrotic area and oxidative substances as well as improving liver functions and impaired motor functions (Figures 4–6, Table 1). These data further confirmed the therapeutic potential of 3-genes iPSCs/iPSC-Heps in AHF.

CCl_4_ is a haloalkane that possesses a hepatotoxic effect. The mechanism of CCl_4_-mediated hepatotoxicity involves reductive dechlorination of carbon tetrachloride (CCl_4_) to a trichloromethyl radical (^•^CCl_3_) which subsequently precipitates membrane lipid peroxidation and hence causes liver damage [13]. TAA, a thiono-sulfur containing compound, has been used as a fungicide, organic solvent, and accelerator in the vulcanization of rubber, as well as a stabilizer of motor oil [19]. TAA administration can lead to accumulation of the reactive oxygen species (ROS) and lipid peroxides, liver cell damage, fibrosis and/or cirrhosis [20–22]. In the livers, the nuclear factor kappa B (NFκB) is consequently activated by TAA administration, followed by elevation of the expression of proinflammatory cytokines [23,24]. Our previous study indicated that engrafted 3-genes iPSCs and 3-genes iPSC-Heps exhibited remarkable ROS scavenging activity in the damaged areas of TAA-injured livers, and protected the liver from TAA-elicited hepatotoxicity [12]. Moriya *et al*. reported that the graft of undifferentiated ESCs can develop into hepatocyte-like cells with appropriate integration into the CCl_4_-injured liver and subsequent improved liver damage [25]. Kuo *et al*. reported that cell therapy using mesenchymal stem cells rescued hepatic failure in CCl_4_-treated mice, probably due to reduction of oxidative stress and accelerated repopulation of hepatocyte after liver damage [26]. In the present study, our data showed that both 3-genes iPSCs and 3-genes iPSC-Heps possess several antioxidant enzymes that protect these cells from CCl_4_-induced death. These cells also showed marked hepatoprotective efficacy on the experimental AHF model induced by CCl_4_ administration. The hepatoprotective activity of 3-genes iPSCs/3-genes iPSC-Heps remained uncertain in CCl_4_-challenged livers. Although the free radical species generated by TAA and CCl_4_ were not fully identical, it is likely that reduction of *in vivo* oxidative stress serves as one of the underlying mechanisms mediating the hepatoprotective effect of 3-genes iPSCs/iPSC-Heps. Hepatic functions were gradually recovered in both MEF and PBS-treated recipients, indicating tissue repair at injury sites (Table 1). Importantly, transplantation of either 3-genes iPSCs or 3-genes iPSC-Heps further improved hepatic functions by facilitating tissue repair at injury sites (Table 1). These data suggested that 3-genes iPSCs/3-genes iPSC-Heps possess a paracrine effect that may stimulate hepatocyte proliferation, and this property may also partially mediate the hepatoprotective effect of 3-genes iPSCs/3-genes iPSC-Heps in CCl_4_-treated mice.

It was extensively demonstrated that stem cells can mobilize into circulation in response to injury signals and contribute to tissue repair at injury sites [26–28]. Hence, the route of cell transplantation is critical for the hepatoprotective activity of 3-genes iPSCs/3-genes iPSC-Heps. Portal vein recruited blood from the gastrointestinal tract and spleen to the liver. Our recent study demonstrated intravenous transplantation of an equal amount of cells led to lower engraftment of 3-genes iPSC-Heps, compared with that of 3-genes iPSCs. Nevertheless, intrasplenic transplantation led to an even extent of hepatic cell engraftment [12]. The discrepancy in the efficacy of intravenous transplantation and intrasplenic transplantation was attributed to the lower mobilization of differentiated cells from the systemic circulation to the injured site, compared with that of undifferentiated cells [12,26]. Intraperitoneal injection is the injection of a substance, preferably in a large amount, into the peritoneum [29]. Lukas *et al*. demonstrated that compounds administered intraperitoneally are absorbed primarily through the portal circulation and must pass thorough the liver before reaching other organs [29]. In the present study, intraperitoneal transplantation also led to an even extent of hepatic engraftment between recipients of 3-genes iPSCs, 3-genes iPSC-Heps, and MEFs, consistent with Lukas *et al.*’s observations addressing the consequence of intraperitoneal administration. These results indicated that the intraperitoneal approach is an ideal route for cell transplantation and could be used for iPSC-based therapy in subsequent researches.

Hepatic encephalopathy, the occurrence of confusion, altered level of consciousness and coma, is one of the complications associated with fulminant hepatic failure [1]. Our findings revealed that 3-genes iPSC-based therapy not only improved hepatic functions and animal survival, but also improved CCl_4_-induced hepatic encephalopathy (Figure 6A). The pathogenesis of hepatic encephalopathy is quite different and the exact mechanisms leading to this functional disturbance are not clearly understood [30]. There is accumulating evidence that a number of pathophysiological mechanisms exist or co-exist, such as elevation of ammonia levels, changes in GABA-receptor complex, changes in serotonin metabolism, storage or release, *etc*. [30]. To our knowledge, this is the first report showing that 3-genes iPSC transplantation improved both AHF and hepatic encephalopathy. This potential therapeutic effect of 3-genes iPSCs against hepatic encephalopathy might be secondary to the normalization of hepatic functions such as the ammonium metabolism. However, future investigations are necessary to elucidate the precise mechanism of the iPSC-mediated effect on hepatic encephalopathy.

## 3. Materials and Methods

### 3.1. 3-Genes iPSC Culture and *in Vitro* Hepatic Differentiation

Murine iPSCs were generated from mouse embryonic fibroblasts (MEFs) derived from 13.5-day-old embryos of C57/B6 mice. The iPSCs were reprogrammed by the transduction of retroviral vectors encoding three transcription factors (Oct4/Sox2/Klf4), as described previously with some modifications [11]. Briefly, undifferentiated iPSCs were routinely cultured and expanded on mitotically-inactivated MEFs (50,000 cells/cm^2^) in six-well culture plates (BD Technology) in the presence of 0.3% leukemia inhibitory factor in an iPSC medium consisting of Dulbecco’s Modified Eagle’s Medium (DMEM; Sigma) supplemented with 15% fetal bovine serum (FBS; Invitrogen), 100 mM minimal essential medium (MEM) nonessential amino acids (Sigma), 0.55 mM 2-mercaptoethanol (Gibco), and antibiotics (Invitrogen). The mouse iPSCs were transfected with pCX-EGFP to constitutively express green fluorescence and were maintained and differentiated *in vitro* as described previously [31]. Every three to four days, colonies were detached with 0.2% collagenase IV (Invitrogen), dissociated into single cells with 0.025% trypsin (Sigma-Aldrich) and 0.1% chicken serum (Invitrogen) in PBS, and replated onto MEFs. For embryoid body (EB) formation, iPSCs were dissociated into a single cell suspension by 0.25% trypsin-EDTA and plated onto non-adherent culture dishes in DMEM with 15% FBS, 100 mM MEM nonessential amino acids, 0.55 mM 2-mercaptoethanol, and antibiotics at a density of 2 × 10^6^ cells/100 mm plate. After 4 days in floating culture, EBs were transferred onto gelatin-coated plates and maintained in the same medium for 24 h. EBs were then assigned for *in vitro* hepatocyte differentiation by using a two-step procedure as previously described, with some modifications [32]. For endoderm induction, iPSCs were incubated for 24 h in RPMI 1640 medium (Invitrogen/Gibco, Rockville, MD, USA), supplemented with 100 ng/mL Activin A (Peprotech). On the following 2 days, 0.1 and then 1% insulin-transferrin-selenium (Invitrogen/Gibco) was added to this medium. Following Activin A treatment, the differentiated iPSCs were cultured in Hepatocyte Culture Medium (HCM) (Cambrex, Baltimore, MD, USA) containing 30 ng/mL FGF4 for 4 days. Then, the differentiated cells were incubated in HCM containing 20 ng/mL HGF for 6 days, in HCM containing 10 ng/mL oncostatin-M (R&D, Minneapolis, MN, USA) plus 0.1 μM dexamethasone (Sigma-Aldrich) for 5 days.

### 3.2. Cellular Uptake Assay of Low-Density Lipoprotein (LDL)

The uptake capability of 1,1′-dioctadecyl-3,3,3′,3′-tetramethylindocarbocyanine perchlorate conjugated to acetylated-LDL (DiI-Ac-LDL; AbD Serotec) of iPSCs and differentiated cells was determined by fluorescent microscopy. Cells were incubated with 20 μg/mL DiI-AC-LDL at 37 °C for 24 h. Incorporation of DiI-Ac-LDL into cells was visualized by fluorescence microscopy.

### 3.3. Periodic Acid-Schiff (PAS) Stain for Glycogen

Cells were fixed in 4% paraformaldehyde, and then permeabilized with 0.1% Triton X-100 for 10 min. Samples were then oxidized in 1% periodic acid for 5 min, rinsed 3 times in deionized water (dH_2_O), treated with Schiff’s reagent for 15 min, and rinsed in dH_2_O for 5 to 10 min. Samples were counterstained with Mayer’s hematoxylin for 1 min and then rinsed in dH_2_O. Samples were observed under the light microscope.

### 3.4. ELISA Analysis

For the determination of the enzyme activities of the liver enzyme cytochrome P_450_ 3A4 and 1A2, cells were collected and properly prepared for analysis of the activities of liver enzyme cytochrome P450 3A4 and 1A2 by a commercialized cytokine assay kit (Promega) according the manufacture’s instructions. The activity of individual cytochrome P450 enzymes was quantified by densitometry and expressed as fold change relative to undifferentiated iPSCs.

### 3.5. Real-Time Reverse Transcription-Polymerase Chain Reaction

Real-time RT-PCR was performed as previously described [33]. For real-time RT-PCR analysis, the total RNA of cells was extracted by using the RNAeasy kit (Qiagen, Valencia, CA, USA). Briefly, the total RNA (1 mg) of each sample was reversely transcribed in 20 mL using 0.5 mg of oligo dT and 200 U Superscript II RT (Invitrogen, Carlsbad, CA, USA). The amplification was carried out in a total volume of 20 mL containing 0.5 mM of each primer, 4 mM MgCl_2_, 2 mL LightCycler FastStart DNA Master SYBR green I (Roche Diagnostics, Pleasanton, CA) and 2 mL of 1:10 diluted cDNA. The quantification of the unknown samples was performed by LightCycler Relative Quantification Software, version 3.3 (Roche Diagnostics). In each experiment, the GAPDH housekeeping gene was amplified as a reference standard. PCR reactions were prepared in duplicate and heated to 95 °C for 10 min followed by 40 cycles of denaturation at 95 °C for 10 s, annealing at 55 °C for 5 s, and extension at 72 °C for 20 s. All PCR reactions were performed in duplicate. Standard curves (cycle threshold values *versus* template concentration) were prepared for each target gene and for the endogenous reference (GAPDH) in each sample. The expression level of liver specific genes was evaluated as the ratio of its mRNA to that of β-actin.

### 3.6. Immunofluorescence Staining

An avidin-biotin complex-based method was used for immunohistochemical staining of differentiated iPSCs. Following washes with 3% hydrogen peroxide, sodium azide and antigenicities were retrieved using a microwave. Each slide was then treated with antibodies for HNF-3β (Chemicon International, Temecula, CA), Albumin (Chemicon International, Temecula, CA, USA), AFP (Upstate Biotechnology, Waltham, MA, USA). Immunoreactive signals were detected with a mixture of biotinylated rabbit anti-mouse IgG and Fluoresave (Calbiochem, La Jolla, CA, USA) and a confocal microscope (Olympus, FV300). The mean numbers of immunoreactive cells of per 100 cells (%) were used in statistical analysis.

### 3.7. Cell Viability Assay

iPSCs were seeded on 24-well plates at a density of 2 × 10^4^ cells/well in medium and submitted to a methyl thiazol tetrazolium assay (MTT assay; Sigma-Aldrich). iPS cells were incubated with 0.25 mg/mL MTT for 4 h at 37 °C, and the reaction was terminated by the addition of 100% isopropanol (16). The amount of MTT formazon product was determined by using a microplate reader, and absorbance was measured at 560 nm (SpectraMax 250, Molecular Devices, Sunnyvale, CA, USA).

### 3.8. Cell-Labeling Protocol

Hoechst 33258 (Sigma) is widely used to label DNA, and these fluorescent stains are commonly used to visualize nuclei. In serum-free culture medium, 1 × 10^6^ human iPSCs were incubated with Hoechst 33258 (10 μM) for 60 min at 37 °C, and then centrifuged at 1500 rpm for 5 min at 37 °C. Later, the supernatant was removed and the cells gently re-suspended in PBS. Hoechst-labeled-iPSCs were directly applied to animal experiments. Fluorescence-labeled cells were observed under a fluorescent microscope. Each slide was evaluated by two separate investigators in a blinded manner. To quantify the scattered density of the incorporated cells, a total of 10 liver sections of each sample were counted on each slide at 100× magnification. The scattered density was expressed as cell count per liver section field in mean ± SD.

### 3.9. Animal Model of Liver Injury

Male BALB/c nude mice, 8 weeks old and weighing 25–30 g, were used for our experiments. Fulminant hepatic failure was induced by intraperitoneal injection of CCl_4_ (2.5 mL/kg body weight; 1:4 v/v in mineral oil). Four hours after CCl_4_ administration, mice were injected with MEFs, iPSCs or iPSC-Heps at 4 different doses: 5 × 10^6^ cells/kg body wt (Group I), 2 × 10^6^ cells/ kg body wt (Group II), 5 × 10^5^ cells/kg body wt (Group III), or 2 × 10^5^ cells/kg body wt (Group IV), via intraperitoneal injection. In order to observe prolonged hepatic damage, measurement of survival and collection of blood samples were performed at 24, 48, or 72 h after the administration of CCl_4_ in recipients of PBS, MEFs, iPSCs, or iPSC-Heps. All mice were caged at 24 °C with a 12-h light-dark cycle and allowed free access to water and food. This study was approved by Taipei Veterans General Hospital Animal Committee, and the principles of Laboratory Animal Care were followed. To explore the role of CCl_4_-elicited reactive oxygen species (ROS) production and evaluate the antioxidant property of *N*-acetylcysteine (NAC), mice were treated chronically with NAC at a dose of 500 mg/kg/day in the drinking water, as previously described [34].

### 3.10. Histological Quantification of Liver Injury

The harvested livers were fixed, embedded in paraffin and sectioned for evaluation of the degree of liver injury. The livers were fixed in 4% paraformaldehyde, dehydrated using graded ethanol, and then embedded in paraffin. The paraffin blocks were sectioned and stained with hematoxylin and eosin (HE) using standard histological techniques. CCl_4_ injection resulted in liver parenchyma damage mainly around the central veins. The necrotic area in an injured liver can be distinguished from normal liver tissue by the brighter color and the presence of inflammatory cell infiltrates. The liver sections were examined and photo-taken under microscope at 10× magnification. The necrotic areas of the injured liver were determined by measuring five independent fields per liver using a computerized morphometry system. The percentage of relative necrotic area (%) was calculated by dividing the necrotic area by total observed area.

### 3.11. Liver Functional Tests

Biochemical parameters were measured using standard clinical methods. After anesthesia by ketamine (10 mg/100 g), intracardiac aspiration of blood was performed. A 0.8–0.9 mL blood sample was collected from the heart in a pyrogen-free syringe containing ~75 units of heparin sodium, then placed in an ice bath and transported immediately to the laboratory. Serum biochemistry tests, including alanine aminotransferase (ALT), aspartate aminotransferase (AST), and total bilirubin, were measured by Vitro DT chemistry system (Johnson & Johnson).

### 3.12. Determination of Intracellular Reactive Oxygen Species (ROS) Production

The measurement of intracellular reactive oxygen species (ROS) production by the probe 2′,7′-dichlorofluorescein diacetate (DCFH-DA; Molecular Probes, Eugene, OR, USA) has been mentioned previously [35]. In brief, cells were incubated with 5 μmol/L DCFH-DA in a culture medium for 30 min at 37 °C, followed by washing with PBS and flow cytometry analysis.

### 3.13. Malondialdehyde Assay

Malondialdehyde (MDA) in liver tissue was assayed using a commercial kit for thiobarbituric acid reactive substances (TBARS; Cayman Chemical, Ann Arbor, MI). Absorbance at 530 nm was determined using an enzyme-linked immunosorbent assay (ELISA) reader (Bio-Rad Laboratories, Hercules, CA, USA).

### 3.14. Nitrate/Nitrite Concentration

Nitrite and nitrate are the primary oxidation products of NO subsequent to reaction with oxygen and, therefore, the nitrite/nitrate concentration was used as an indicator of NO synthesis. Nitrite/nitrate levels in mouse tissue were measured after enzymatic conversion of nitrate to nitrite using nitrate reductase. Subsequently, total nitrite in liver tissue was assayed by adding 100 μL Griess reagent (0.05% naphthalethylenediamine dihydrochloride and 0.5% sulphanilamide in 2.5% phosphoric acid) to each sample. The optical density at 550 nm (OD550) was measured and the total nitrite/nitrate concentration for each sample was calculated by comparison of the OD550 of a standard solution of sodium nitrate prepared in saline.

### 3.15. Measurement of Motor Activity

Motor activities in an open field were determined by using the Opto-Varimex animal activity meter (Columbus Instruments Inc.) [36,37]. The Opto-Varimex activity sensors utilize high-intensity, modulated infrared light beams to detect animal motion. Animals were housed in transparent cages (17 inches × 17 inches × 8 inches) through which 30 infrared beams pass in the horizontal plane, 15 on each axis. This device differentiates non-ambulatory movements (scratching, gnawing) from ambulation on the basis of consecutive interruption of the infrared monitoring beams. An additional row of infrared beams in the horizontal plane (15 on each axis) about 10 cm above the floor was used to count the vertical movements. During the activity measurements, animals have no access to food or chow. All studies were performed under strictly standardized conditions in the dark room for 30 min. The counting numbers of the total movements, ambulatory movements, and vertical movements were separately recorded to reflect the motor activities of rats with fulminant hepatic failure. The motor activities were defined as zero in dead mice.

### 3.16. Statistical Analysis

Results were expressed as mean ± S.D. Statistical analyses were performed by using unpaired Student’s *t*-tests, and the survival rate analysis using log-rank test. Results were considered statistically significant at *P* < 0.05.

## 4. Conclusions

In conclusion, this report demonstrated that iPSCs and iPSC-Heps expressing only Oct-4/Sox2/Klf-4 effectively rescued CCl_4_-induced AHF, via an intraperitoneal route of cell transplantation. The hepatoprotective effect of 3-genes iPSCs/iPSC-Heps may be partially through their antioxidant activity and/or facilitating the hepatic tissue repair in a paracrine manner. Considering the prominent hepatoprotective effect of the 3-genes iPSCs/iPSC-Heps in different models of fulminant hepatic failure, these 3-genes iPSCs/3-genes iPSC-Heps may serve as an alternative against AHF.

## Figures and Tables

**Table 1:** Effect of intraperitoneal transplantation of mouse embryonic fibroblasts (MEFs), iPSC-Heps, or induced pluripotent stem cells (iPSCs) on hepatic biochemical parameters in CCl_4_-treated recipients.

	Alanine aminotransferase (ALT; IU/L)	Aspartate aminotransferase (AST; IU/L)	Total bilirubin (TBIL; mg/dL)
**BALB/c nude mice**	11.1 ± 3.5	55.2 ± 10.8	0.45 ± 0.4
**Recipient day 1 Post CCl** **_4_**			
*PBS*	10125.5 ± 680.5	6610.4 ± 800.5	4.40 ± 0.42
*MEFs*	9912.2 ± 620.0	5618.9 ± 428.2	4.24 ± 0.12
*iPSC-Heps*	5084.5 ± 432.8 *	2359.5 ± 307.1 *	3.24 ± 0.22 *
*iPSCs*	2339.3 ± 349.8 *,#	1369.3 ± 210.0 *,#	2.28 ± 0.20 *,#
**Recipient day 2 Post CCl** **_4_**			
*PBS*	3135.1 ± 451.4	2660.6 ± 286.5	3.11 ± 0.35
*MEFs*	2679.8 ± 424.7	2241.2 ± 249.4	2.22 ± 0.21
*iPSC-Heps*	1807.6 ± 305.5 *	1527.5 ± 179.8 *	1.90 ± 0.28 *
*iPSCs*	865.6 ± 171.2 *,#	767.5 ± 227.4 *,#	1.29 ± 0.15 *,#
**Recipient day 3 Post CCl** **_4_**			
*PBS*	598.0 ± 60.2	412.1 ± 28.5	2.18 ± 0.17
*MEFs*	459.2 ± 56.1	362.8 ± 8.5	1.32 ± 0.12
*iPSC-Heps*	139.1 ± 10.9 *	104.5 ± 8.8 *	1.22 ± 0.18 *
*iPSCs*	95.6 ± 5.5 *,#	60.3 ± 6.7 *,#	1.13 ± 0.17 *,#

Results were expressed as mean ± SD from ten recipients.

* *P* < 0.05 *vs*. MEFs;

# *P* < 0.05 *vs*. iPSC-Heps.

**Table 2:** Incidence of tumor formation in recipients of 4-genes iPSCs, 3-genes iPSCs, or 3-genes iPSC-Heps four months after transplantation.

Group tumor formation	1 month	2 months	3 months	4 months
4-genes iPSCs	0/6	1/6	2/6	3/6
3-genes iPSCs	0/6	0/6	0/6	0/6
4-genes iPSC-Heps	0/6	0/6	0/6	0/6
