# Correction: Chang et al. Improvement of Carbon Tetrachloride-Induced Acute Hepatic Failure by Transplantation of Induced Pluripotent Stem Cells Without Reprogramming Factor c-Myc. *Int. J. Mol. Sci.* 2012, *13*, 3598–3617

**DOI:** 10.3390/ijms27125403

**Published:** 2026-06-16

**Authors:** Hua-Ming Chang, Yi-Wen Liao, Chih-Hung Chiang, Yi-Jen Chen, Ying-Hsiu Lai, Yuh-Lih Chang, Hen-Li Chen, Shaw-Yeu Jeng, Jung-Hung Hsieh, Chi-Hsien Peng, Hsin-Yang Li, Yueh Chien, Szu-Yu Chen, Liang-Kung Chen, Teh-Ia Huo

**Affiliations:** 1 Department of Optics and Photonics, National Central University, Chung-Li 32001, Taiwan; huamingchang@gmail.com (H.-M.C.); sychen@dop.ncu.edu.tw (S.-Y.C.); 2 Institute of Oral Biology, School of Dentistry, National Yang-Ming University, Taipei 11217, Taiwan; rabbity18@yahoo.com.tw (Y.-W.L.); henlichen@nycu.edu.tw (H.-L.C.); 3 Institute of Pharmacology, National Yang-Ming University, Taipei 11217, Taiwan; guchiang@gmail.com (C.-H.C.); ylchang@vghtpe.gov.tw (Y.-L.C.); 4 Division of Urology, Department of Surgery, Taipei Veterans General Hospital, Su-Ao/Yuan-Shan Branch, Yilan County 26444, Taiwan; jsy202@mail.ysvh.gov.tw (S.-Y.J.); alainjhh@yahoo.com.tw (J.-H.H.); 5 School of Medicine, National Yang-Ming University, Taipei 11217, Taiwan; chenyj@vghtpe.gov.tw (Y.-J.C.); d49405004@gmail.com (Y.-H.L.); chpeng1008@gmail.com (C.-H.P.); lihy@vghtpe.gov.tw (H.-Y.L.); lkchen2@vghtpe.gov.tw (L.-K.C.); 6 Department of Obstetrics and Gynecology, Taipei Veterans General Hospital, Taipei 11217, Taiwan; 7 Department of Medical Research and Education, Taipei Veterans General Hospital, Taipei 11217, Taiwan; 8 Department of Ophthalmology, Shin Kong Wu Ho-Su Memorial Hospital, Taipei 11101, Taiwan; 9 School of Medicine, Fu-Jen Catholic University, Taipei 24352, Taiwan; 10 Center for Geriatrics and Gerontology, Taipei Veterans General Hospital, Taipei 11217, Taiwan; 11 Division of Gastroenterology, Department of Internal Medicine, Taipei Veterans General Hospital, Taipei 11217, Taiwan

In the original publication [1], there was a mistake in Figure 5B as published. The authors occasionally found that the original 5B contains unintentional misplacements. The corrected Figure 5 appears below. The authors state that the scientific conclusions are unaffected. This correction was approved by the Academic Editor. The original publication has also been updated.

## Figures and Tables

**Figure 5:**
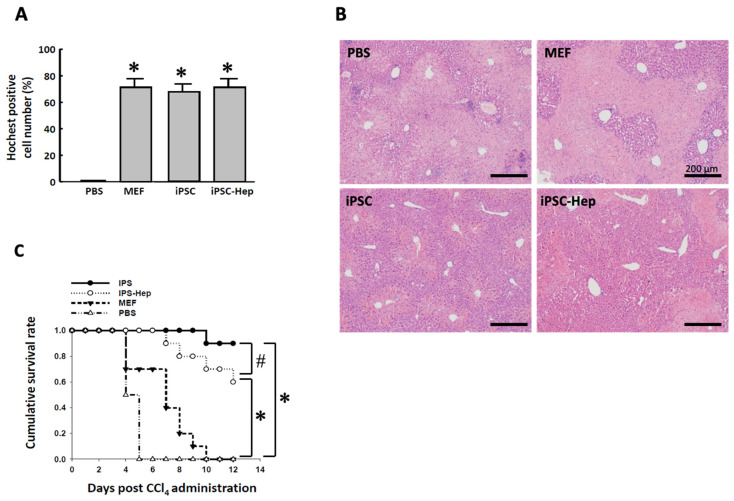
Effect of intraperitoneal cell transplantation (PBS, MEFs, 3-genes iPSCs, or 3-genes iPSC-Heps) on hepatic pathology, survival rate and biochemical parameters in CCl_4_-treated recipients. (**A**) Hoechst-stained cell engraftment in the CCl_4_-injured liver from recipients of PBS, MEFs, iPSCs, or iPSC-Heps; (**B**) Representative H & E stain of CCl_4_-treated liver tissue 24 h after receiving iPSCs, iPSC-Heps, MEFs or PBS treatment; (**C**) Intraperitoneal transplantation of 3-genes iPSCs or 3-genes iPSC-Heps rescued mice from lethal AHF. Data shown here are the mean ± SD of six independent experiments. In panel, * *p* < 0.05 vs. PBS. In panel C, * *p* < 0.05 vs. PBS or MEFs. # *p* < 0.05 vs. iPSC-Heps.
